# Closure of Oro-Antral Communication Using Buccal Advancement Flap

**DOI:** 10.29252/wjps.8.2.262

**Published:** 2019-05

**Authors:** Ritul Patel, Prutha Patel, Viral Kalariya, Het Patel, Chetan Chavda

**Affiliations:** Thunder Bay Regional Health Sciences Centre, Thunder Bay, Canada

**Keywords:** Oro-antral, Closure, Flap, Fistula

## Abstract

Improper and inadequate treatment can lead to oro-antral communication and fistula. Certain surgical procedure during operation in posterior maxilla can lead to communication between oral cavity and sinus. In children and adolescents, the risk of oro-antral communication is less, due to smaller volume of the maxillary sinus defect smaller than 2 mm that would adequately heal without any intervention, but larger communications more than 2 mm would require immediate attention from surgeon and treatment should be done as soon possible in order to avoid further complications, infection and patient’s discomfort.

## INTRODUCTION

The maxillary sinus is the largest part into upper jaw known as antrum of Higmore, as first defined by anatomist Higmore as space in the bone and labeled as antrum in 1651.^1^ An oroantral complication, frequently happens in a dental office. Oroantral communication is an abnormal passage between maxillary sinus and oral cavity. Most frequent cause of this complication happens during extraction of maxillary molars and premolars up to 48%. The anatomic reason is most likely the culprit here, that being anatomic proximity or projection of roots within the sinus.^2^ Sinus mucosa thickness varies between 1 and 7 mm as reported by Skoglund *et al.*^3^ Tuberosity fracture, implant dislodgement, trauma, cyst and tumors in the maxillary sinus, osteoradionecrosis, flap necrosis, dehiscence after implant, periapical infection, tuberosity fracture are other reason of oroantral communication/oro-antral communication and fistula.^2^^,^^4^^,^^5^

Without any infection in sinus defects smaller than 2 mm, can heal following blood clot formation via the secondary healing. Larger defects than those left untreated, likely to take a course of acute sinusitis, almost half of the patients within 2 days of time, and that likely to progress up to 90% within 14 days.^4^^,^^6^ Closure of this defect is at stake to prevent any food or saliva accumulation which causes contamination and leads to infection delay or impaired healing and chronic sinusitis.^7^ There are many techniques for the closure of oroantral communication including buccal or palatal alveolar flaps and their modifications. The preferred technique depends on surgeons past experience and expertise.

## CASE REPORT

**Fig. 1:**
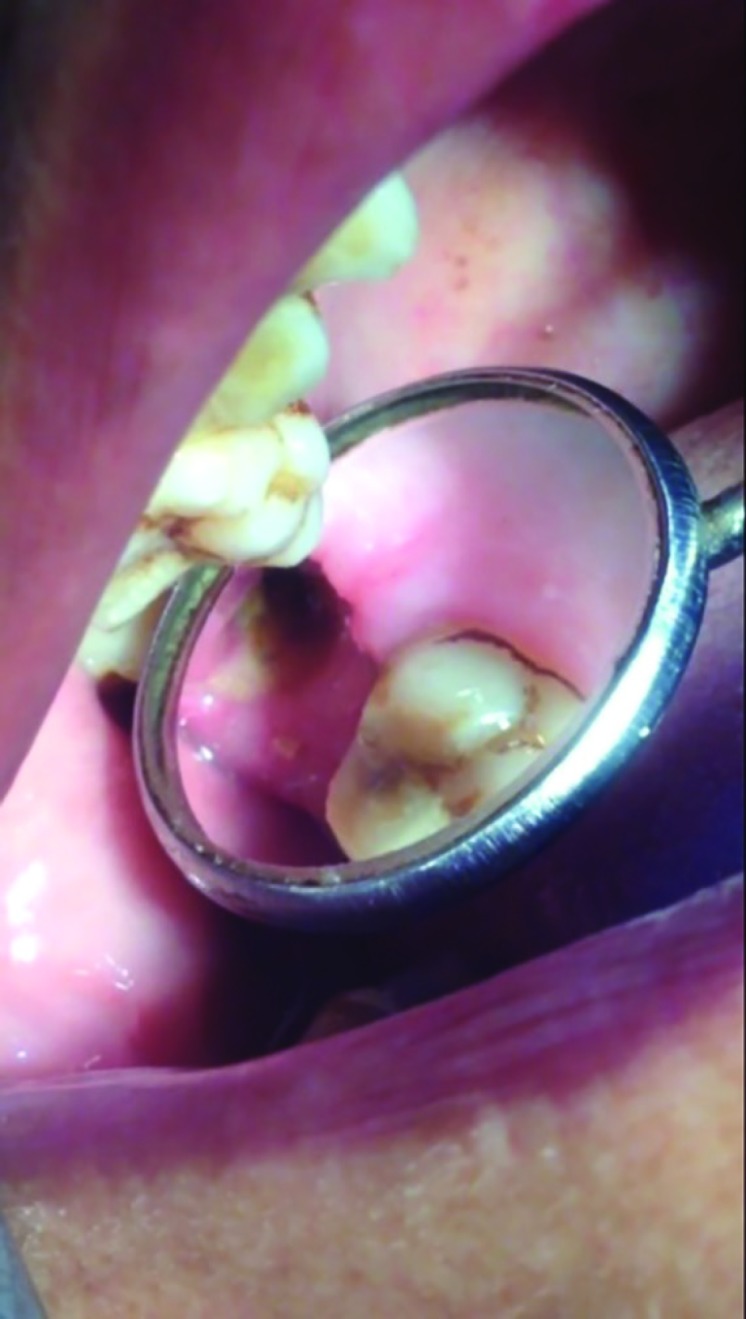
The oroantral communication in the patient

A 40 years old female was presented with a chief complaint of nasal regurgitation, pain, and halitosis. Relevant dental history was taken, which revealed extraction of maxillary right second molar, 7 days back. Clinical investigation and mirror fog test were done to confirm the oroantral communication (Figure 1). The patient was made aware of condition and treatment plan and all risk of surgery and was started on preoperative medications. Surgery was planned for the next day. Under local anesthesia, buccal advancement flap with a trapezoidal shape and two vertical releasing incisions were elevated. 

**Fig. 2:**
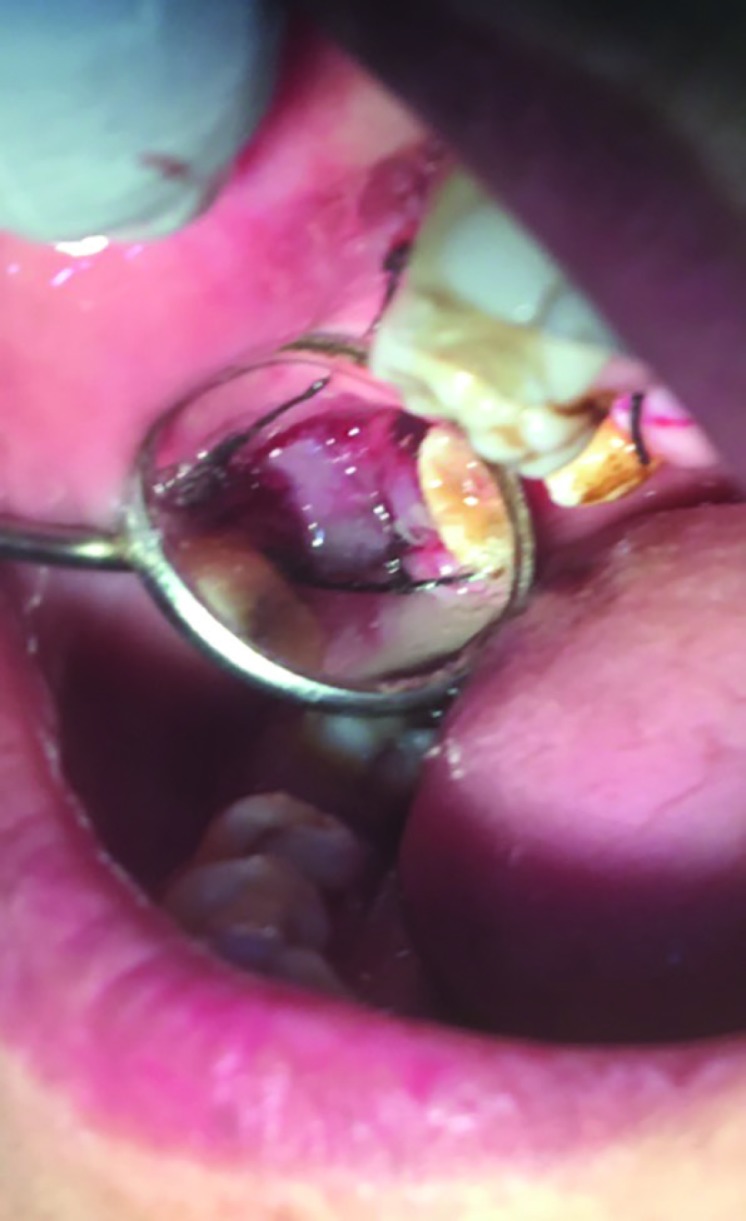
The palatal flap was raised to suture buccal flap

**Fig. 3:**
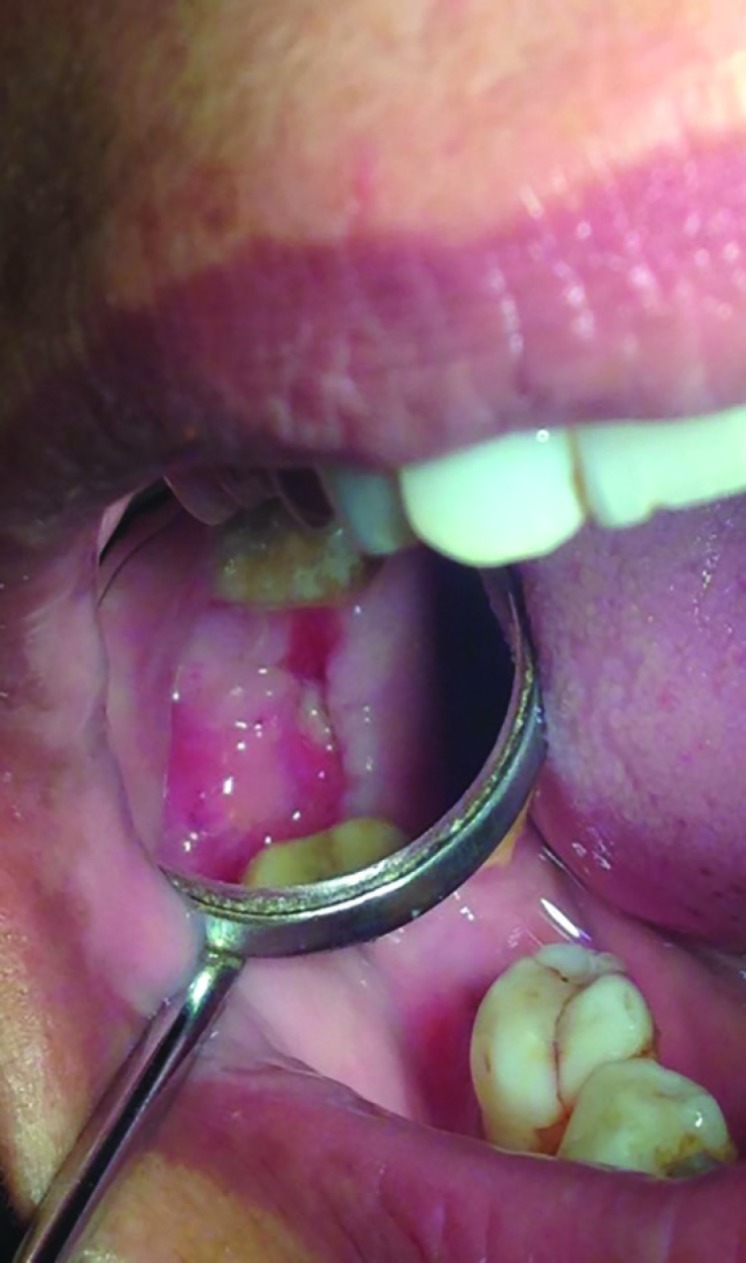
Healing without nasal regurgitation.flap

The periosteal scoring method was used to make flap tension free at closure site, which was crucial into this type of procedure leading to a higher success ratio of surgery. The socket was irrigated with 0.12% chlorhexidine gluconate solution and socket lining was removed fresh bleeding induced into the socket. The palatal flap was also raised to suture buccal flap, easily. The buccal flap was sutured using 3.0 silk over a socket to palatal flap (Figure 2). Postoperative instructions and medication were advised and scheduled for suture removal after 10 days. Healing was as desired and uneventful with no nasal regurgitation and pain (Figure 3). 

## DISCUSSION

All different parameters should be taken in light, when deciding surgical closure including the location, size of oroantral communication, alveolar ridge height, sinus inflammation and patient’s overall condition. All flaps used for closure of oroantral communication or oroantral communication and fistula are relied on mobilizing tissue flap and tension free closure on the defect. When the defect was located on mesial, a buccal flap is more likely indicated.^8^ The only disadvantage of this technique is the loss of vestibule, which in an era of a dental implant that may not be much of concern or sometimes it requires vestibuloplasty in denture bearing patient. Two thumb rule is a must for oroantral communication or oroantral communication and fistula to (i) make infection free, whatever is there or to make sure it is infection free, and (ii) to have tension free closure for any soft tissue flap being used. A different technique of using bone blocks and bone graft to preserve the site for future implant placement was suggested nowadays by many doctors again for the chance of any infection, delayed healing, graft failure and flap necrosis that all should be carefully addressed.^8^

Various methods are described in the literature including use of various techniques and different material and approaches, but the main goal should be emphasized for proper closure of the defect. Success also is dependent upon patient co-operation for following the strict post-operative instructions and not creating any kind of negative pressure in the mouth. All necessary medications should be prescribed to avoid any pain, discomfort or infection during recovery. 

## CONFLICT OF INTEREST

The authors declare no conflict of interest.

## References

[1] Highmore N (1651). Corporis humani disquisitio anatomica.

[2] Hassan O, Shoukry T, Raouf AA, Wahba H (2012). Combined palatal and buccal flaps in oroantral fistula repair. Egyptian Journal of ENT and Allied Sciences.

[3] Skoglund LA, Pedersen SS, Holst E (1983). Surgical management of 85 perforations to the maxillary sinus. Int J Oral Surg.

[4] Scattarella A, Ballini A, Grassi FR, Carbonara A, Ciccolella F, Dituri A, Nardi GM, Cantore S, Pettini F (2010). Treatment of oroantral fistula with autologous bone graft and application of a non-reabsorbable membrane. Int J Med Sci.

[5] Khandelwal P, Hajira N (2017). Management of Oro-antral Communication and Fistula: Various Surgical Options. World J Plast Surg.

[6] Haas R, Watzak G, Baron M, Tepper G, Mailath G, Watzek G (2003). A preliminary study of monocortical bone grafts for oroantral fistula closure. Oral Surg Oral Med Oral Pathol Oral Radiol Endod.

[7] Borgonovo AE, Berardinelli FV, Favale M, Maiorana C (2012). Surgical options in oroantral fistula treatment. Open Dent J.

[8] Amaratunga NA (1986). Oro-antral fistulae--a study of clinical, radiological and treatment aspects. Br J Oral Maxillofac Surg.

